# Jiao-tai-wan for insomnia symptoms caused by the disharmony of the heart and kidney: a study protocol for a randomized, double-blind, placebo-controlled trial

**DOI:** 10.1186/s13063-020-04299-x

**Published:** 2020-05-15

**Authors:** Congcong Zeng, Xi Liu, Lufeng Hu, Yuan Feng, Nengzhi Xia, Haihuan Zeng, Lin Luo, Ren Ye, Zhengzhong Yuan

**Affiliations:** 1 Department of Traditional Chinese Medicine, The First Affiliated Hospital of Wenzhou Medical University, Nanbaixiang, Ouhai District, Wenzhou, 325035 Zhejiang China; 2 Zhejiang Chinese Medical University, Binwen Road, Binjiang District, Zhejiang, 310053 Hangzhou China; 3 Department of Pharm, The First Affiliated Hospital of Wenzhou Medical University, Wenzhou, 325035 Zhejiang China; 4 The 2nd Clinical College of Wenzhou Medical University, Chashan Higher Education Park, Wenzhou, 325035 Zhejiang China; 5 X-ray Department, The First Affiliated Hospital of Wenzhou Medical University, Wenzhou, 325035 Zhejiang China; 6 Sleep monitoring center, The First Affiliated Hospital of Wenzhou Medical University, Wenzhou, 325035 Zhejiang China; 7 Research Institute of China Academy of Chinese Medical Sciences, No.16 Nanxiao Street, Dongzhimen, Dongcheng District, Beijing, 100700 China

**Keywords:** Jiao-tai-wan, Insomnia, Traditional herbal medicine, Randomized controlled trial, Study protocol, Pattern identification

## Abstract

**Background:**

Insomnia seriously affects people’s normal lives and work. However, effective treatment strategies are scarce. The purpose of this study is to explore the efficacy and safety of Jiao-tai-wan (JTW) for ameliorating insomnia symptoms caused by disharmony of the heart and kidney.

**Design:**

This is a randomized, double-blind, placebo-controlled pilot clinical trial. A total of 124 participants suffering from insomnia symptoms will be randomly assigned to the JTW or placebo group in an equal ratio. The participants will be asked to take JTW or placebo granules twice a day for 1 week. All data will be gathered at baseline and at the end of the drug intervention. The primary outcome measures will be the mean change in the Pittsburgh Sleep Quality Index (PSQI) from baseline to the end of the drug intervention. Secondary outcome measures will include the altered sleep parameters in polysomnography, ^1^H-magnetic resonance spectroscopy (^1^H-MRS) evaluation, the Disharmony of Heart and Kidney Scoring System score, and blood tests, including the levels of serum adenosine and melatonin. A laboratory test will be taken before and after treatment to assess the safety of JTW.

**Discussion:**

The outcomes of this study will confirm the efficacy of JTW for the treatment of insomnia symptoms and will also be used to monitor the safety of JTW.

**Trial registration:**

Chinese Clinical Trial Registry, ChiCTR1800019239. Registered on 1st November 2018.

## Background

Sleep is essential to human health, but unfortunately, nearly one-third to one-quarter of the population in resource-poor countries suffer from insomnia at some level [1, 2]. With the increasing incidence of insomnia, there are different degrees of depression, anxiety, and other psychological diseases, which seriously affect the quality of a person’s daily life [3]. Psychological and behavioral therapy, Western medicinal therapy, and traditional medicinal therapy are the main treatments for insomnia [4]. Many clinical studies have supported the first two treatments; however, some risks of adverse reactions and tolerance have been reported. In addition, dependence and addiction seriously impact clinical efficacy. For instance, the long-term use of zolpidem, which is relatively safe and frequently prescribed for the treatment of transient insomnia, will increase the risk of dementia [5]. Thus, interest in using traditional medicinal therapies is increasing, especially in traditional herbal medicine [6].

The superiority and soul of traditional East Asian medicine is the system called “pattern identification,” which is used to diagnose and treat diseases based on the symptoms and the signs observed in patients. According to this system, insomnia has many different patterns, but disharmony of the heart and kidney is the dominant pattern [7, 8]. Moreover, Jiao-tai-wan (JTW) has been commonly used for the management of insomnia with a disharmony of heart and kidney pattern for centuries in China, Korea, and Japan [9].

In a review article by Yeung et al. [10], JTW was listed as one of the 10 most frequently examined standardized traditional herbal formulas for insomnia. A clinical study conducted in China showed that administration of JTW for 60 consecutive days could improve the Pittsburgh Sleep Quality Index (PSQI) [11]. Lu et al. [12] reported a significant decrease in scores in the Disharmony of Heart and Kidney Scoring System. Another study [13] demonstrated the effects of JTW in detail by alleviating the symptoms of palpitation and dizziness caused by the disharmony of the heart and kidney.

Although recent clinical studies have shown the efficacy of JTW in the treatment of insomnia, a randomized, double-blind, placebo-controlled pilot clinical trial to confirm its efficacy has not yet been undertaken. We set up this study to better clarify the efficacy, safety, and feasibility of JTW for treating insomnia caused by disharmony of the heart and kidney and to provide data for evidence-based clinical practice.

## Methods and design

### Study design

**Fig. 1:**
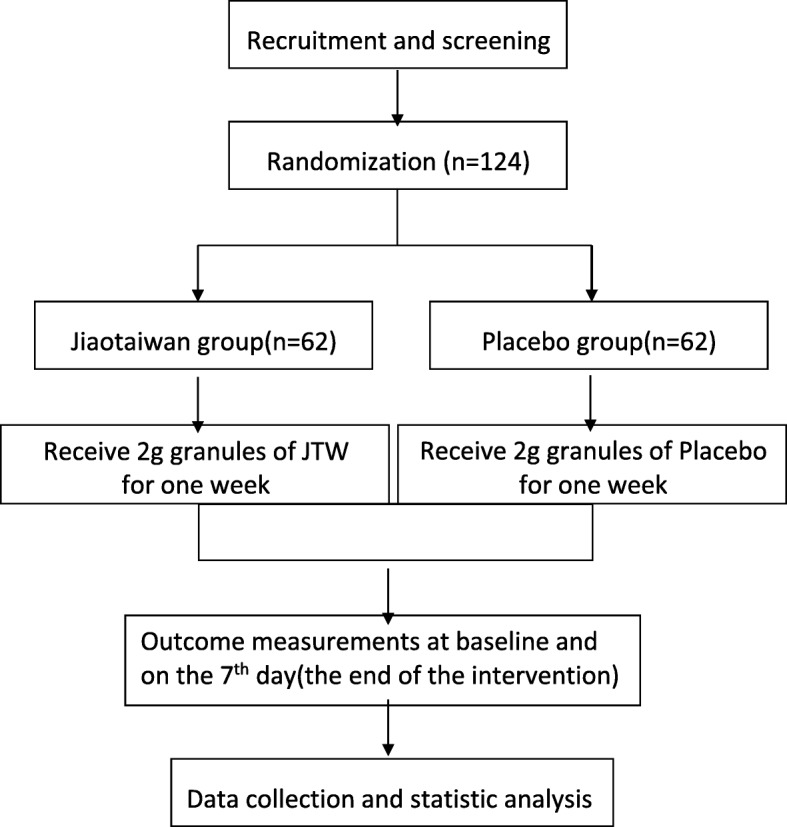
Flow chart of the study design

**Fig. 2:**
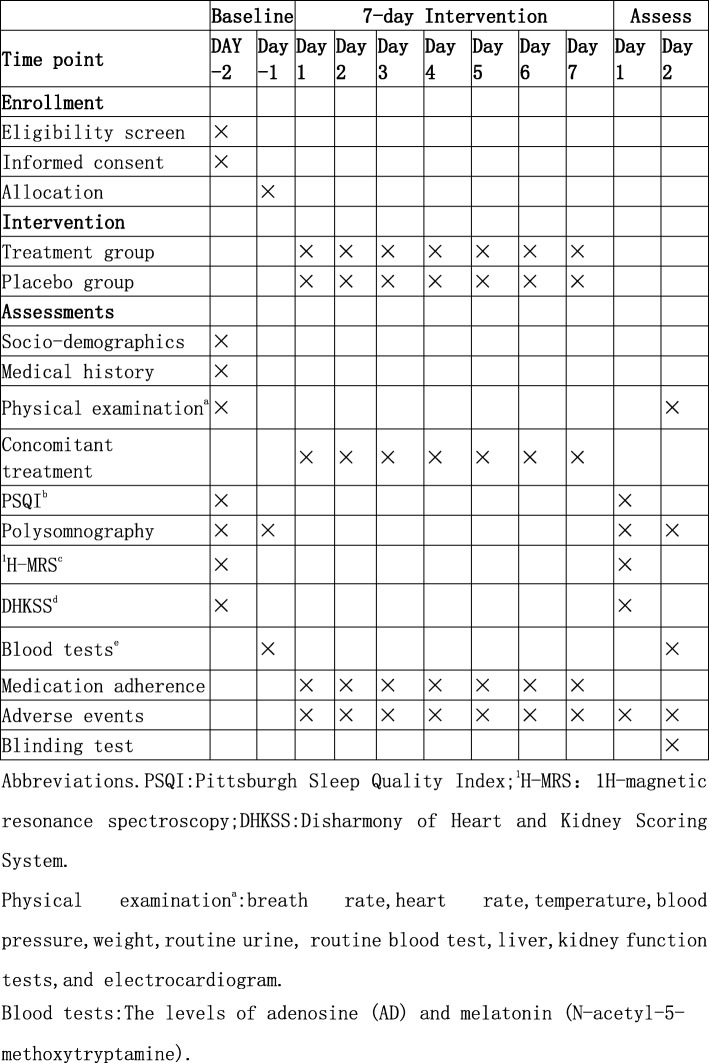
SPIRIT schedule for enrollment, treatment, and assessments

This is a parallel group randomized superiority clinical trial comparing JTW to placebo for efficacy in treating insomnia. The choice of using a placebo for the trial in order to test the rationality of the study design and to explore the effectiveness of JTW. Eligible participants will be randomly assigned to either the JTW or placebo group in an equal proportion. The drug intervention will last for 1 week. Figure 1 briefly shows the study flow chart and Fig. 2 enumerates the treatment schedule and outcome measures. The study adheres to the Standard Protocol Items: Recommendations for Interventional Trials (SPIRIT 2013) checklist [14] (Additional file 1).

### Participants

#### Study population

Patients suffering from insomnia symptoms caused by the disharmony of the heart and kidney will be recruited from the Traditional Chinese Medicine Department and Sleep Centre of the First Affiliated Hospital of Wenzhou Medical University in Wenzhou, Zhejiang Province, China. To recruit target participants, advertisements will be distributed using flyers, Wechat, and online and offline bulletin boards of the hospital.

#### Inclusion criteria

1. Participants are aged 18–60 years, either male or female, and with a level of education above junior high school.
2. Insomnia is almost the only symptom, including difficulty sleeping, deep sleep, dreaminess, early-morning awakening, trouble going back to sleep, feeling tired after waking up, or daytime sleepiness (excluding secondary insomnia).
3. A PSQI score of 7 or higher according to the Chinese Classification and Diagnostic Criteria of Mental Disorders-3 Version, CCMD-3 [15].
4. A Disharmony of Heart and Kidney Scoring System score of 9 or higher.
5. Signed informed consent before treatment.

#### Exclusion criteria

1. Insomnia caused by physical diseases or lifestyle changes or environmental disturbances.
2. Affective disorders, anxiety disorder, depression, schizophrenia, and any other serious mental disorders.
3. Suffering from somatic diseases that affect the central nervous system
4. Serious heart, liver, kidney, and hematopoietic system diseases, abnormal liver and kidney function.
5. Regular use of antipsychotic drugs in the last month.
6. Use of medicine to treatment sleeping disorders in the past week.
7. Allergic to a certain ingredient of the drug involved in this trial or suffering from allergies.
8. Alcohol abuse and/or psychotropic drug addiction.
9. Pregnancy or lactation.

### Randomization and allocation concealment

We will apply block randomization to generate a sequence of labels (A or B) at 1:1 ratio, numbered from 001 to 124. This randomization approach forces balance between treatment and placebo groups over time as the recruitment proceeds. The pharmaceutical company will prepare the trial intervention packages according to the labeled sequence (A or B), numbered from 001 to 124, all in uniform plastic packages. The study coordinator will be blinded to the group assignment and will give participants the numbered package of intervention according to their visiting time sequence at baseline visit (e.g. the first participant will get the package numbered 001, the second participant will get packaged 002, and so on). Thus, the allocation concealment will be maintained throughout the study. The generated sequence of the group assignment and numbers will be sealed in opaque envelopes and stored in double-locked cabinets. Allocation concealment will be maintained throughout the entire study.

### Blinding

The present study will use a double-blind method. The placebos and JTW will look the same and will be packaged in uniform plastic bags. Then, the study coordinator will assign JTW or placebo according to the randomization codes and place them in special containers. The participants, outcome assessors, study coordinators, data managers, and statisticians will be blinded to the group allocation and numbered drug containers throughout the entire study. At the end of the study, the blinding codes will be revealed.

If unblinding is required, the trial managers and data coordinators will have access to group allocations and any unblinding will be reported.

### Intervention

These eligible participants will ingest 2 g granules of JTW or placebo, twice a day at 1600 hours and 2100 hours for 1 week and present themselves at the termination of medication, followed by a scheduled examination.

Both JTW and placebo are manufactured by the Kangren Pharmaceutical Factory according to good manufacturing practice standards. JTW will be produced in the form of granules with a yellowish-brown color. The medication will be 2 g, containing 1.1 g JTW soft extract with 0.37 g of lactose hydrate and 0.88 g of corn starch as the excipient. The JTW soft extract will be made of a water extract of a mixture of two herbal medicines, as follows: Rhizome Coptidis (10 g) and Cortex Cinnamomi (1 g). The placebo will consist of corn starch, lactose hydrate, citric acid, and caramel color. The placebo and JTW will have the same appearance and color.

### Medication compliance monitoring

To ensure compliance with the medication, participants will be asked to count the actual intake and return the empty wrapping papers for the medication and any remaining medicine.

### Prohibition and permission for concomitant treatment

1. The use of any sedative hypnotics to help sleep will be prohibited during the study.
2. The use of any traditional medicine designed to treat insomnia, except for the intervention of this trial, will be prohibited during the study.
3. The use of functional foods or other medications intended to improve the symptoms of insomnia will be prohibited during the study.
4. Any psychotherapy for accelerating sleep quality will be prohibited during the study.
5. Medications used to treat other chronic diseases at the beginning of this trial will be allowed.
6. Routine physical training before the start of this clinical trial will be allowed.
7. Any change in medications or physical exercise during the study will be recorded in a clinical report form.

### Outcome measurement

#### Primary outcome

We chose 1 week to assess the curative effect of JTW based on: (1) our previous experimental results and findings on animal sedation and hypnosis [16]; and (2) our clinical experience with patients using JTW.

##### Pittsburgh Sleep Quality Index

The PSQI will be used at the baseline and the end of the drug intervention. The PSQI version used in this study is a 19-item self-reported retrospective questionnaire to access the quality of sleep in the past 7 days, and it is designed to measure seven domains called component scores: subjective sleep quality; sleep latency; sleep duration; habitual sleep efficiency; sleep disturbances; use of sleep medications; and daytime dysfunction [16, 17]. Component scores range from 0 (no difficulty) to 3 points (severe difficulty), and when summed, produce a global score in the range of 0–21. A higher score denotes worse sleep. These syndromes are categorized as mild (0–1), moderate (2–7), or severe (≥ 8) [18]. Then, the scores the participants obtained at baseline will be recorded as score 0, and the scores obtained at the end of drug intervention will be recorded as score 1. The recorder will calculate the reduction rate (RR) according to the following formula: RR = (score 1–score 0)/score 0*100%. Finally, we will assess the clinical curative effect of every participant based on the results of the RR. RR values > 75% will be regarded as a clinical cure, RR values between 50%–75% will be considered as a clinical effective, RR values between 25%–50% will be recognized as a clinical success, and RR values < 25% will be considered ineffective. The primary outcome is defined to have a value of 0 if RR is < 25% (ineffective) and 1 if RR is > 25% (clinical cure, clinically effective, or clinical success). The proportion of 1s (i.e. RR ≥ 25%) will be compared between the treatment and placebo groups.

In addition, Liu et al. [18] presented their opinion that the PSQI is suitable for Chinese patients after they conducted reliability and validity tests [19].

#### Secondary outcomes

##### Polysomnography

Polysomnography (PSG) will be taken twice, at both the baseline and the end of the drug intervention. On the first night, participants will adapt themselves to the laboratory environment. On the second night, participants will be placed on PSG monitoring to record the multiple physiological sleep parameters. The parameters [20, 21] will include total sleep time, sleep efficiency, sleep latency, rapid eye movement (REM) stage latency, wake after sleep onset, and the time duration of the particular sleep stages (such as N1, N2, N3). The mean changes of all the parameters from the baseline to the end of the drug intervention will be used to measure the changes in sleep quality.

PSG is considered the gold standard for scoring sleep disorders. The scoring of sleep and arousals relies on visual inspection of continuous surface electroencephalography (EEG), electromyography (EMG), and electrooculography (EOG) during PSG and then divides human sleep into five stages: wakefulness (W); REM; non-rapid eye movement (NREM); and N1, N2, and N3 [22]. The American Academy of Sleep Medicine (AASM) defined the quantitative reference standard in PSG for adult insomnia as follows [23]: sleep latency ≥ 30 min; total sleep time < 390 min; number of wake after sleep onset (WASO) ≥ 2 or time of WASO ≥ 40 min; time in N1/total sleep time > 60%; time in N2 / total sleep time > 60% or time in N3/total sleep time < 10%, or time in REM / total sleep time < 20%. A higher or lower score corresponds to more severe symptoms.

##### ^1^H-magnetic resonance spectroscopy

^1^H-magnetic resonance spectroscopy (^1^H-MRS) will also be taken at the baseline and the end of the drug intervention. ^1^H-MRS, with its unique non-invasive advantages, is able to detect and quantify the important metabolites of living brain tissue, including N-acetylaspartate (NAA), choline (Cho), creatine (Cr), gamma-aminobutyric acid (GABA), and myo-intositol (mI) [24]. In the present study, single voxel hippocampus and thalamus metabolite ratios of GABA with Cr will be measured. The altered ratios of GABA with Cr between baseline and the end of the drug intervention will be the second outcome measure.

Studies have revealed that the GABA/Cr ratio in the frontal lobe is significantly lower [25], and the average brain GABA levels are nearly 30% lower in patients with primary insomnia [26].

##### Disharmony of Heart and Kidney Scoring System

The Disharmony of Heart and Kidney Scoring System will be used at the baseline and the end of drug intervention. It is a checklist covering one indispensable and seven accompanying items. These items are the symptoms and signs of the disharmony of heart and kidney pattern according to the theory of the pattern identification system in traditional East Asia medicine. The score of the indispensable item uses a 4-point scale (0, 3, 6, and 9) depending on the severity of the insomnia (0 = none, 9 = very severe). The seven accompanying items including palpitations, dizziness, spermatorrhea or menstrual irregularity, and night sweat and use scale of 0–4 points based on their frequency. A total score of > 9 out of 30 points is thought to demonstrate disharmony of the heart and kidney [27, 28].

Although there is no accurate evidence to explain the validity and reliability of this questionnaire, no other methods are currently available to identify the disharmony of heart and kidney pattern.

##### Blood tests

Blood tests consists of the levels of adenosine (AD) and melatonin (N-acetyl-5- methoxytryptamine) in blood samples will be recorded at the baseline and the end of the drug intervention and will then be analyzed by the clinical lab.

Sleep homeostasis in adults is affected by the sleep-regulatory substances AD and melatonin [29, 30]. AD is a product of brain metabolism and is closely related to sleep parameters. A previous study [31] showed that AD levels were elevated as a consequence of sustained wakefulness. Melatonin is an endogenous hormone produced by the pineal gland and is released exclusively at night. Melatonin has been shown to synchronize the circadian rhythms, and improve the onset, duration, and quality of sleep. Takaesu et al. thought that reduced secretion of melatonin may be involved in the mechanism of insomnia [32].

In the present study, high-performance liquid chromatography (HPLC) will be developed to determine the levels of adenosine, and an enzyme-linked immunosorbent assay will be used to detect the concentration of melatonin.

### Safety outcomes

At the end of this trial, participants will participate in a routine physical examination consists of breath rate, heart rate, temperature, blood pressure, weight, routine urine, routine blood test, liver, kidney function tests, and electrocardiogram.

### Reporting and treating of adverse events

During the treatment period, the researchers will record any adverse events (AEs) that will be defined as unpredictable, undesirable symptoms, signs, or diseases related to the treatment during daily telephone calls. The researchers will then comprehensively evaluate the correlation between these AEs and the experimental drugs according to the recorded details about AEs, including the manifestation, occurrence time, duration, cause, and treatment measures. Simultaneously, if any AE occurs, the investigator will take proper measures such as dose adjustment, drug withdrawal, and symptomatic treatment to ensure the safety of participants; all details will be written down carefully. Furthermore, continuous follow-up will be insisted upon until the condition of the participants returns to normal. Furthermore, an insurance contract will be concluded to cover financial compensation to participants who might be injured during the study.

### Participant drop-outs

1. The participant can decide to discontinue treatment at any time for any reason.
2. The patient is non-compliant.
3. The patients experiences a serious AE.

Drop-outs and withdrawals from the trial will be recorded throughout the intervention and follow-up periods. Their data will be collected in the case report form but will not be used for the final statistical analysis.

### Sample size

The ratio of cases between the JTW group and the placebo group is set to 1:1; this study is designed to achieve a statistical power of 80% (two-sided type-1 error of *α* = 5%, *β* = 20%). Based on previous clinical practice [33, 34], we assume a treatment success rate of 40% in the placebo group and 70% in the JTW group over 7 days. The sample size is calculated using the following formula:

$$ N={\left[{\Phi}^{-1}\left(\alpha \right)+{\Phi}^{-1}\left(\beta /2\right)\right]}^2\times \left({\pi}_e+{\pi}_0\right)\left(100-\left({\pi}_e+{\pi}_0\right)/2\right)/{\left({\pi}_e-{\pi}_0\right)}^2 $$

where Φ is the cumulative distribution function of the standard normal distribution, and *π*_*e*_ and *π*_0_ are the success rate in the experimental and control groups, respectively. Considering a 20% drop-out rate, a sample size of *N* = 49/(1–0.2) = 62 is needed for each group.

Thus, under the assumptions of a success rate of 70% for treatment and 40% for placebo, and a non-compliance rate of 20%, the sample size required to have at least 80% power of detecting a difference between the two groups at a two-sided alpha level of 5% is 124 participants.

### Data management and monitoring

The experimental data will be carefully saved in Microsoft Access, a database management system (DBMS) from Microsoft. To guarantee the data quality, data will be input and checked twice by two researchers. During the study, the First Affiliated Hospital of Wenzhou Medical University is responsible for making regular visits (once a month) to review trial conduct; the Ethics Committee will monitor for protocol violations monthly. There was no conflict of interest with the sponsors or researchers.

In addition, confidentiality of participants’ data is ensured by using participants’ IDs rather than identifiable information in the dataset (i.e. coding) and by storing the document linking the IDs to the identifiable information separately and securely. Only researchers directly involved in the analysis of the randomized controlled trial (RCT) will have access to the final trial dataset, which will only contain coded data. After data collection and before data storage, all outcomes are manually double-checked by the research staff. The safety, progress, study integrity, and design aspects will be monitored at various meetings by the research team involved in this study.

### Statistical analysis

We will use two-sided *p* values for the primary outcome and for all secondary outcomes and safety variables. For the primary outcome, we will use Fisher’s exact test to test the hypothesis at null: the treatment has the same efficacy as the placebo. For each secondary outcome, defined as the value difference between baseline and end-of-intervention, we will use the following tests: (1) t-test for the continuous PSG variables; Chi-square test for the categorical PSG variables; (2) t-test for the blood-level adenosine and melatonin; (3) t-test for the GABA/Cr ratio; and (4) Wilcoxon rank sum test for the Disharmony Score of Heart and Kidney. For safety evaluation of JTW, a two-sided t-test will be applied to each safety measurement after data normalization.

We define the analysis population as the enrolled participants who have successfully completed the trial. We will characterize the types of individuals who have missing data to better understand the nature the missing mechanisms. We will use statistical models to impute missing values if the assumption of missing at random is met. This can preserve and utilize the information of the non-missing values.

## Discussion

JTW, first appearing in an old classical text of ancient Chinese medicine [35], is a well-known tranquillizing formula to treat insomnia due to disharmony of the heart and kidney [36]. As mentioned, modern scientific studies have shown that JTW may be helpful for people suffering from insomnia [10–13]; however, to the best of our knowledge, this is the first RCT that will determine the efficacy and safety of JTW in the therapy of insomnia symptoms, which is why we intend to perform this study.

However, the present study has some limits. First, the study period is short, with short time follow-up times. Second, the pattern of insomnia in this study is relatively simple, with only the pattern of disharmony of the heart and kidney included. Lastly, a single-center sample study is less convincing than a multi-center sample study. Despite these limits, the present study is reasonably well designed. The results of the present study are expected to guide physicians or traditional medical doctors at clinics more clearly in prescribing JTW and to widely spread the use of traditional medicine in other countries.

## Trial status

Protocol version date and number: February 10, 2020; version 1.3.

Patient recruitment began in September 2018 and it will be completed by December 2020.

## Supplementary information

**Additional file 1.** SPIRIT checklist.

## Data Availability

The datasets used or analyzed during the current study will be available from the corresponding author upon reasonable request.

## Abbreviations

AASM: American Academy of Sleep Medicine
AD: Adenosine
AE: Adverse event
CCMD-3: Chinese Classification and Diagnostic Criteria of Mental Disorder-3 Version
Cho: Choline
Cr: Creatinine
EEG: Electroencephalography
EMG: Electromyography
EOG: Electrooculography
GABA: Gamma-aminobutyric acid
^1^H-MRS: ^1^H-magnetic resonance spectroscopy
HPLC: High performance liquid chromatography
JTW: Jiao-tai-wan
mI: Myo-inositol
NAA: N-acetylaspartate
NREM: Non-rapid eye movement
PSG: Polysomnography
PSQI: Pittsburgh Sleep Quality Index
RCT: Randomized controlled trial
REM: Rapid eye movement
RR: Reducing rate
SPIRIT: Standard Protocol Items: Recommendations for Interventional Trials
W: Wakefulness
WASO: Wake after sleep onset
